# Supplementary material for: Neuroprotective Effect of Bcl-2 on Lipopolysaccharide-Induced Neuroinflammation in Cortical Neural Stem Cells
Source: Int J Mol Sci. 2022 Jun 7;23(12):6399. doi: 10.3390/ijms23126399 (PMC9223771; doi:10.3390/ijms23126399)
Supplement: Supplementary file 1 [file ijms-23-06399-s001.zip › ijms-1732452-supplementary.pdf]

Supplementary Information

Neuroprotective effect of Bcl-2 on lipopolysaccharide-induced neuroinflammation in cortical neural stem cells

Shin-Young Park and Joong-Soo Han

Biomedical Research Institute and Department of Biochemistry and Molecular Biology, College of Medicine, Hanyang University, 222 Wangsimni-ro, Seongdong-gu, Seoul 04763, Republic of Korea.

Co-correspondence

Shin-Young Park. Tel.: 82-2-2220-0610; Fax: 82-2-2220-2422; E-mail: ttokttoki@hanyang.ac.kr

Joong-Soo Han. Tel.: 82-2-2220-0623; Fax: 82-2-2220-2422; E-mail: jshan@hanyang.ac.kr

Supplementary Figure S1

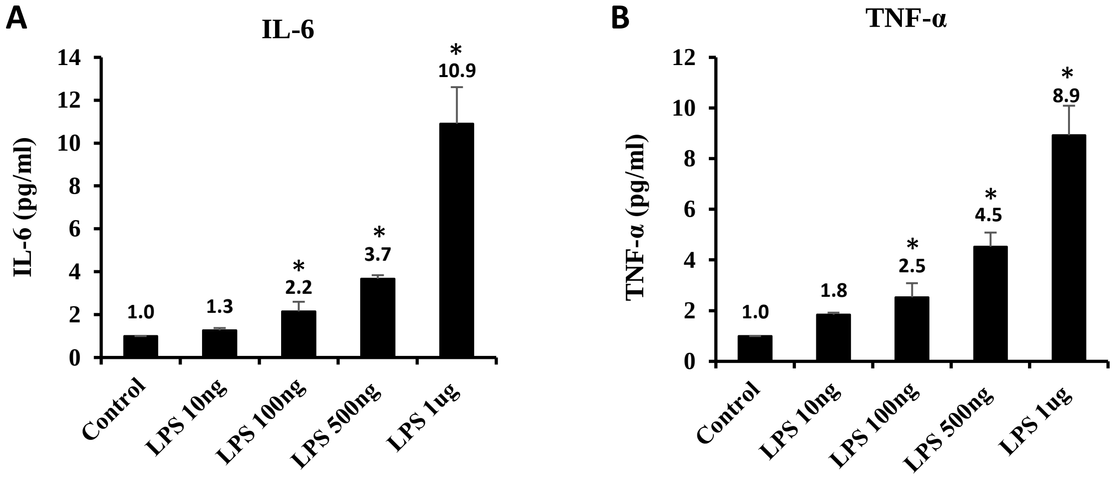

Supplementary Figure S1. Pro-inflammatory cytokine production in LPS-treated NSCs. (A and B) NSCs were treated with LPS at 10 ng, 100 ng, 500 ng, or 1 μg/mL for 12 h in the absence of bFGF. The mRNA levels of *IL-6* and *TNF-α* were analyzed by real-time RT-PCR; $n = 3$. Data are shown as mean ± SD. * $p < 0.05$ compared with control, for *IL-6* and *TNF-α* respectively. Statistical significances were assessed by one-way ANOVA with a post hoc Tukey's test.

Supplementary Figure S2

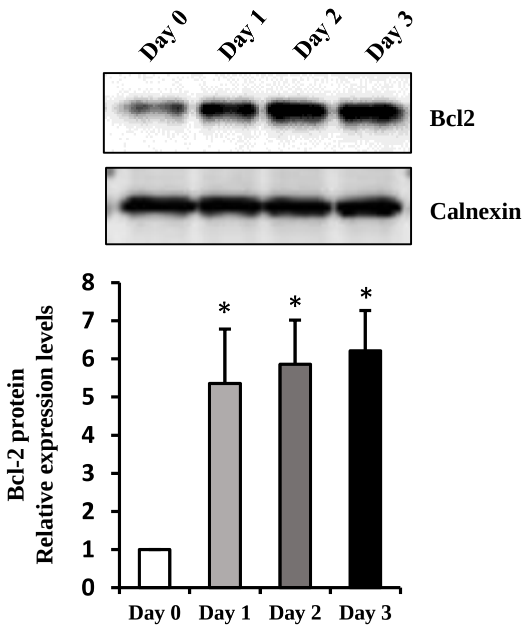

Supplementary Figure S2. Bcl-2 expression during neuronal differentiation in NSCs. NSCs were differentiated for 1, 2 or 3 days in the absence of bFGF, respectively. Western blotting was performed using anti-Bcl-2 or anti-calnexin antibodies to detect the respective protein bands (upper panel). Band intensity (graph) was quantified with Quantity Ones® software. Data are shown as mean ± SD. * $p < 0.05$ compared with Day 0. Statistical significances were assessed by one-way ANOVA with a post hoc Tukey's test.
